# The prognostic significance of lymphovascular invasion in patients with resectable gastric cancer: a large retrospective study from Southern China

**DOI:** 10.1186/s12885-015-1370-2

**Published:** 2015-05-07

**Authors:** Peng Li, Hao-Qiang He, Chong-Mei Zhu, Yi-Hong Ling, Wan-Ming Hu, Xin-Ke Zhang, Rong-Zhen Luo, Jing-Ping Yun, Dan Xie, Yuan-Fang Li, Mu-Yan Cai

**Affiliations:** 1 State Key Laboratory of Oncology in South China, Collaborative Innovation Center for Cancer Medicine, Sun Yat-sen University Cancer Center, Guangzhou, China; 2 Department of Pathology, Sun Yat-sen University Cancer Center, No. 651, Dongfeng Road East, 510060 Guangzhou, China; 3 Diagnostic Imaging and Intervening Center, Sun Yat-sen University Cancer Center, Guangzhou, China; 4 Department of Gastric & Pancreatic Surgery, Sun Yat-sen University Cancer Center, No. 651, Dongfeng Road East, 510060 Guangzhou, China

**Keywords:** Lymphovascular invasion, Prognosis, Gastric cancer

## Abstract

**Background:**

The focus of this study was to assess the impact of lymphovascular invasion (LVI) on both the recurrence of cancer and the long-term survival of Chinese patients with resectable gastric cancer (GC).

**Methods:**

A retrospective analysis of the clinicopathological data for 1148 GC patients who had undergone gastrectomy with regional lymphadenectomy was performed. The primary objective was to assess the correlation between LVI and post-surgery outcomes for each patient. This was done by routine H & E staining for LVI on patients’ disease-free survival (DFS) and disease-specific survival (DSS).

**Results:**

LVI was detected in 404 (35.2%) of the 1148 GC patients. The presence of LVI was significantly correlated with the level of CA19-9, the tumor size, the Lauren classification, tumor differentiation, gastric wall invasive depth, lymph node involvement, distant metastasis and an advanced TNM stage. There was a lower DFS and DSS in the patients with LVI as compared to the patients without LVI. A multivariate analysis also identified LVI as an independent prognostic factor of both DSS and DFS.

**Conclusions:**

The presence of LVI is a risk factor for the recurrence of cancer and an independent indicator of a poor outcome in GC patients following surgery. The LVI status should be taken into consideration when determining the best approach for the treatment of the individual.

## Background

Gastric cancer (GC) continues to be a major challenge in the health care community worldwide especially in East Asian countries; such as China, South Korea and Japan [1,2]. Despite the advances in medical treatments, gastrectomy with regional lymphadenectomy remains the primary treatment for patients with resectable GC and has a five-year overall survival (OS) rate of approximately 20-30%. The low OS rate is due to the high frequency in the post-surgery recurrence of cancer [3,4].

The most reliable indication of the prognosis following surgery can be provided through the assessment of the GC using the International Union Against Cancer/American Joint Committee on Cancer (UICC/AJCC) TNM staging guidelines [5]. However, it has been noted that many patients that have been determined to have the same UICC/AJCC TNM stage have heterogeneous survival rates. Therefore, there has been an increased focus on determining other prognostic indicators that will aid in the identification of GC patients with a higher risk for the recurrence of their cancer and who may be candidates for other adjuvant therapies.

The major factor contributing to the recurrence of cancer and mortality is thought to be the systemic dissemination of cancer cells. Lymphovascular invasion (LVI), also referred to as blood vessel and/or lymphatic invasion, is the presence of tumor cells within the lumen of the blood and/or lymphatics; the process of which leads to circulating tumor cells. The presence of LVI is a common pathological finding in a variety of human cancers and has been shown to be associated with a high recurrence rate and poor prognosis in patients with breast cancer, colorectal cancer, non-small cell lung cancer and clear cell renal cell carcinoma [6-10]. The combination of traditional TNM staging with an assessment for LVI could lead to a more accurate indication of the patient’s prognosis [11].

Previous studies have investigated the prognostic significance of LVI in relation to GC in small selected cohorts. The results of these studies indicated that the presence of LVI, either in the blood or lymphatics, correlated with tumor recurrence and a low survival rate that appeared to be independent of lymph node status [8,12-18]. The prognostic value of an LVI assessment in GC remains controversial due to the small number of participants in the study. To address this issue, a large retrospective study of GC patients who had undergone surgery in Southern China was designed and carried out to thoroughly investigate the correlation between LVI and cancer-recurrence/ long-term survival.

## Methods

### Patient selection

Of the 3321 GC patients that had undergone surgery in Sun Yat-sen University Cancer Center (Guangzhou, China) between May of 1996 and June of 2009, 1148 (34.6%) were selected because they had a gastrectomy with lymphoadenectomy. This was determined using the archives of the Department of Pathology based upon the following criteria: (1) a histologically confirmed primary gastric adenocarcinoma; (2) no neoadjuvant treatment before operation; (3) complete resection of the tumor; (4) resection margins were negative; (5) detailed and complete follow-up data.

Variables included the gender of the patient (female and male), age at the time of surgery (<60 and ≥ 60 years), the level of preoperative serum carcinoembryonic antigen (CEA; elevated and normal), levels of the carbohydrate antigen (CA19-9; elevated and normal), approximate tumor size (≤5 and > 5cm), tumor differentiation (well, moderate and poor), Lauren classification (intestinal, mixed and diffuse), infiltration depth (T1, T2, T3 and T4), lymph node status (N0, N1, N2 and N3), distant metastasis (absent and presence), TNM stage (I, II, III and IV), LVI (absent and presence) and recurrence. Detailed information is given in Table 1. The immunoradiometric method was used to measure the serum concentrations of both CEA and CA19-9. The cut-off values for CEA and CA19-9 were 5.0 ng/ml and 35.0 U/ml; serum concentrations found to be above these respective cut-off values were defined as elevated. In the first year post surgery patients were followed up every three months; the following two years they were seen every six months and annually thereafter. A complete history and physical examination, gastroscopy, gastrointestinal barium examination, CT and MRI was done in order to assess tumor recurrence; which was defined as local recurrence or metastasis. The disease-free survival (DFS) was defined as the time interval post-surgery until recurrence/metastasis or death from gastric cancer (GC), whichever came first. The disease-specific survival (DSS) was defined as the time interval post-surgery until the date of death resulting from GC or the date of the last follow-up exam. This study was approved by the Institute Research Medical Ethics Committee of Sun Yat-sen University Cancer Center. All patient information was hidden to reviewers. No informed consent, written or verbal, was obtained for the retrospective use of the tissue specimens from the patients in this study, however, since most were deceased approval was deemed unnecessary by the Ethics Committee and the need for consent was waived.

**Table 1:** **Correlation between lymphovascular invasion and clinicopathologic characteristics in gastric carcinoma**

Variables	Lymphovascular invasion			
	All cases	Absence	Presence	*P* value^*^
Sex				0.955
Female	354	229 (64.7%)	125 (35.3%)	
Male	794	515 (64.9%)	279 (35.1%)	
Age at diagnosis (years)				0.458
<60	574	378 (65.9%)	196 (34.1%)	
≥60	574	366 (63.8%)	208 (36.2%)	
CEA^†^				0.665
Normal	791	525 (66.4%)	266 (33.6%)	
Elevated	157	107(68.2%)	50 (31.8%)	
CA19-9^‡^				0.004
Normal	707	487 (68.9%)	220 (31.1%)	
Elevated	195	113 (57.9%)	82 (42.1%)	
Size (diameter), cm				<0.0001
≤5	697	487 (69.9%)	210 (30.1%)	
>5	451	257 (57.0%)	194 (43.0%)	
Lauren classification				0.004
Diffuse	585	356 (60.9%)	229 (39.1%)	
Mixed/ Intestinal	563	388 (68.9%)	175 (31.1%)	
Differentiation				<0.0001
Well/moderate	435	331 (76.1%)	104 (23.9%)	
Poor/undifferentiated	713	413 (57.9%)	300 (42.1%)	
Gastric wall invasion				<0. 0001
T1/T2	150	140 (93.3%)	10 (6.7%)	
T3/T4	998	604 (60.5%)	394 (39.5%)	
Nodal metastasis				<0.0001
N0	377	322 (85.4%)	55 (14.6%)	
N1-N3	771	422 (54.7%)	349 (45.3%)	
Distant metastasis				<0.0001
M0	1003	679 (67.7%)	324 (32.4%)	
M1	145	65 (44.8%)	80 (55.2%)	
TNM stage				<0.0001
I/II	486	409 (84.2%)	77 (15.8%)	
III/ IV	662	335 (50.6%)	327 (49.4%)	

^*^Chi-square test; ^†^Preoperative serum CEA was measured in 948 patients; ^‡^Preoperative serum CA19-9 was measured in 902 patients; CEA indicates carcinoembryonic antigen; CA19-9 indicates carbohydrate antigen 19-9.

### Pathological evaluation

Standard pathological procedures were followed in the processing of all surgical specimens. H & E-stained slides of the primary tumors and regional lymph nodes were independently examined by two pathologists; both of whom had no prior knowledge of the clinical parameters of the patient. Discrepancies were resolved through the simultaneous re-examination of the slides using a double-headed microscope by both pathologists. For each tumor, there were at least three slides available for pathological evaluation. The WHO Classification of Tumours of the Digestive System (2010 version) was used to determine tumor differentiation. The depth of tumor infiltration, the lymph node status and the tumor stage was determined utilizing the UICC/AJCC TNM (tumor-node-metastasis) Classification System (2010 version). LVI was defined as the invasion of vessel walls by tumor cells and/or the presence of tumor emboli within an endothelial-lined space; with no distinction between vascular and lymphatic vessels [8]. The following criterion was used to identify the lumen of blood and/or lymph vessels: (i) lined by endothelium; (ii) with supporting smooth muscle or elastica; (iii) filled with lymphatic fluid or red blood cells. Alternative circumstances were considered artifacts due to peritumoral edema and tissue shrinkage.

### Statistical analysis

The Chi-square test was used in order to identify the correlation between LVI and clinicopathologic variables in GC patients. Both DFS and DSS were calculated using the Kaplan-Meier method and the differences between the patient groups were analyzed utilizing a log-rank test in a univariate analysis. A Cox proportional hazard model was utilized for a multivariate analysis in order to determine independent prognostic factors. All tests were two sided and a *P* value of < 0.05 was considered to be statistically significant. Statistical analyses were performed using The SPSS 16.0 statistical software (SPSS, Chicago, IL, USA).

## Results

### Clinicopathologic characteristics in patients with resectable GC

**Figure 1:**
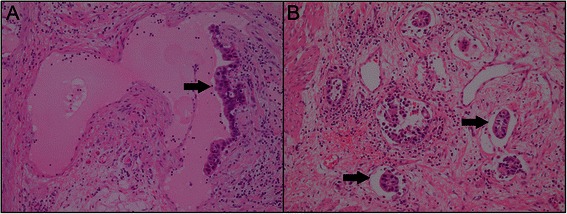
**Histological patterns of lymphovascular invasion in gastric cancer.(A)** Vessel walls were invaded by tumor cells. **(B)** Tumor emboli were observed within an endothelium-lined space.

The clinicopathological features of our GC cohort are detailed in Table 1. A total of 1148 patients; with a male-to-female ratio of 2.24:1, were included in the present study. The median age at the time of resection was 59.0 years (range, 18.0 to 84.0 years). The presence of LVI was detected in 404 patients (35.2%); LVI was identified as the invasion of vessel walls by tumor cells (Figure 1A) and/or the presence of tumor emboli within an endothelial-lined space (Figure 1B).

### The correlation of LVI with clinicopathologic characteristics in patients with resectable GC

The correlation between LVI and clinicopathologic characteristics is shown in Table 1. Our analyses support a significant correlation between the presence of LVI and the level of CA19-9, tumor size, Lauren classification, tumor differentiation, infiltration depth, lymph node involvement, distant metastasis and TNM stage (*P* = 0.004 for CA19-9 level and Lauren classification; *P* < 0.0001 for the others). However, no significant correlation was found between the presence of LVI and other variables; such as gender, age and CEA level (*P* > 0.05).

### The prognostic impact of LVI in patients with resectable GC

**Figure 2:**
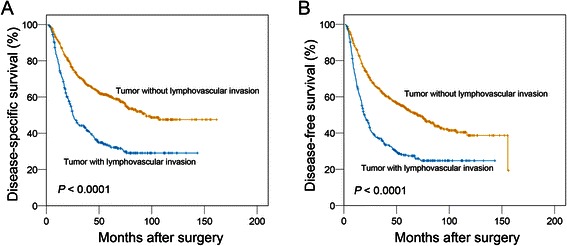
**The impact of lymphovascular invasion on the prognosis of patients with gastric cancer (log-rank test).** There was a statistically significant difference in the disease-specific survival **(A)** and disease-free survival **(B)** between lymphovascular invasion-positive and -negative patients.

**Figure 3:**
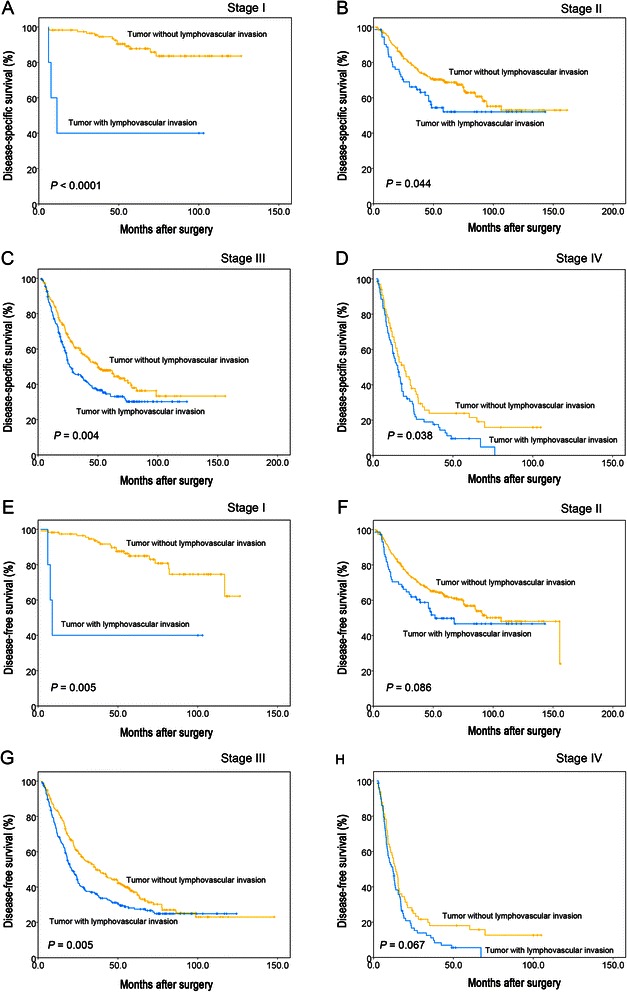
**The prognostic significance of lymphovascular invasion in patients stratified by the TNM stage (log-rank test).** Stage-match survival analysis showed that the presence of LVI was a prognostic factor for DSS in GC patients with stage I, stage II, stage III or stage IV **(A-D)**. Stage-match survival analysis demonstrated that LVI was a statistically significant predictor for DFS in stage I or stage III and a tendency towards statistical significance was found in stage II (*P* = 0.086) or stage IV (*P* = 0.067, **E-H**).

The average time interval between surgery and the follow-up examination was 40.4 months (range, 1.0 to 161.5 months). The five-year DSS and DFS rates for all 1148 patients were 51.0% and 44.6%. The five-year DSS was determined to be 33.1% in patients with LVI and 60.4% in patients without LVI; as determined using the log-rank test analysis which also indicated that there was a significant difference between the two groups (P < 0.0001) (Figure 2A) The analysis also indicated that DFS was significantly decreased in patients with LVI as compared to those without LVI (27.8% vs. 53.1%, *P* < 0.0001) (Figure 2B). A stratified analysis was performed to evaluate the correlative impact of identifying LVI at each TNM stage as it relates to patient survival. Our results indicate that the presence of LVI was a reliable prognostic factor for DSS in GC patients with stage I, stage II, stage III or stage IV (*P* < 0.05, Figure 3A-D). Similar results were obtained when focusing on DFS. LVI was determined to be a reliable indicator of DFS in stage I or stage III (*P* = 0.005 for both) and showed a tendency towards statistical significance when found in stage II (*P* = 0.086) or stage IV (*P* = 0.067), as determined by doing a stage-match survival analysis (Figure 3E-H).

### LVI is an independent predictor of poor outcome in patients with resectable GC

The univariate analysis indicated that certain variables were shown to correlate with DSS; these variables include age at the time of surgery (*P* = 0.046), CA19-9 level (*P* = 0.001), tumor size (*P* < 0.0001), Lauren classification (*P* < 0.0001), tumor differentiation (*P* < 0.0001), infiltration depth (*P* < 0.0001), lymph node metastasis (*P* < 0.0001), distant metastasis (*P* < 0.0001), TNM stage (*P* < 0.0001) and LVI (*P* < 0.0001, Table 2). A Cox proportional hazard model was performed using the multivariate analysis in order to determine independent prognostic factors of DSS. The independent variables shown to correlate with the post-surgical DSS were confirmed to be the tumor size (HR, 1.311; 95%CI, 1.077-1.595, *P* = 0.007), infiltration depth (HR, 2.284; 95%CI, 1.413-3.691, *P* = 0.001), distant metastasis (HR, 2.365; 95%CI, 1.851-3.022, *P* < 0.0001), TNM stage (HR, 2.090; 95%CI, 1.462-2.988, *P* < 0.0001) and LVI (HR, 1.438; 95%CI, 1.171-1.766, *P* = 0.001) (Table 2). Similarly, LVI was found to be an independent prognostic factor for DFS in GC patients after curative resection (HR, 1.393; 95%CI, 1.150-1.688, *P* = 0.001, Table 3).

**Table 2:** **Univariate and multivariate analyses of different prognostic factors in 1148 patients with gastric carcinoma for disease-specific survival**

Variable	Univariate analysis^*^			Multivariate analysis	
	All cases	HR (95% CI)	*P* value	HR (95% CI)	*P* value
Sex			0.800		
Female	354	Reference			
Male	794	0.977 (0.817-1.169)			
Age at diagnosis (years)			0.046	1.170 (0.960-1.425)	0.120
≤59	574	Reference			
>59	574	1.184 (1.003-1.398)			
CEA^†^			0.082		
Normal	791	Reference			
Elevated	157	1.231 (0.974-1.556)			
CA19-9^‡^			0.001	1.098 (0.878-1.374)	0.411
Normal	707	Reference			
Elevated	195	1.438 (1.155-1.791)			
Size (diameter), cm			<0.0001	1.311 (1.077-1.595)	0.007
≤5	697	Reference			
>5	451	1.695 (1.436-2.000)			
Lauren classification			<0.0001	0.836 (0.619-1.129)	0.243
Diffuse	585	Reference			
Mixed/ Intestinal	563	0.740 (0.627-0.875)			
Differentiation			<0.0001	1.126 (0.816-1.554)	0.471
Well/moderate	435	Reference			
Poor/undifferentiated	713	1.445 (1.211-1.724)			
Gastric wall invasion			<0.0001	2.284 (1.413-3.691)	0.001
T1/T2	150	Reference			
T3/T4	998	4.643 (3.083-6.991)			
Nodal metastasis			<0.0001	0.806 (0.573-1.134)	0.216
N0	377	Reference			
N1-N3	771	2.529 (2.061-3.102)			
Distant metastasis			<0.0001	2.365 (1.851-3.022)	<0.0001
M0	1003	Reference			
M1	145	3.479 (2.832-4.272)			
TNM stage			<0.0001	2.090 (1.462-2.988)	<0.0001
I/II	486	Reference			
III/ IV	662	3.039 (2.516-3.670)			
Vascular invasion			<0.0001	1.438 (1.171-1.766)	0.001
Absent	744	Reference			
Present	404	2.121 (1.795-2.506)			

^*^Cox regression model; ^†^Preoperative serum CEA was measured in 948 patients; ^‡^Preoperative serum CA19-9 was measured in 902 patients; HR indicates hazards ratio; CI indicates confidence interval; CEA indicates carcinoembryonic antigen; CA19-9 indicates carbohydrate antigen 19-9.

**Table 3:** **Univariate and multivariate analyses of different prognostic factors in 1148 patients with gastric carcinoma for disease-free survival**

Variable	Univariate analysis^*^			Multivariate analysis	
	All cases	HR (95% CI)	*P* value	HR (95% CI)	*P* value
Sex			0.936		
Female	354	Reference			
Male	794	1.007 (0.851-1.192)			
Age at diagnosis (years)			0.359		
≤59	574	Reference			
>59	574	1.075 (0.921-1.255)			
CEA^†^			0.068		
Normal	791	Reference			
Elevated	157	1.227 (0.985-1.528)			
CA19-9^‡^			<0.0001	1.145 (0.929-1.411)	0.204
Normal	707	Reference			
Elevated	195	1.499 (1.221-1.840)			
Size (diameter), cm			<0.0001	1.334 (1.111-1.602)	0.002
≤5	697	Reference			
>5	451	1.643 (1.407-1.919)			
Lauren classification			0.001	0.821 (0.619-1.090)	0.173
Diffuse	585	Reference			
Mixed/ Intestinal	563	0.768 (0.657-0.897)			
Differentiation			<0.0001	1.085 (0.801-1.470)	0.598
Well/moderate	435	Reference			
Poor/undifferentiated	713	1.374 (1.166-1.618)			
Gastric wall invasion			<0.0001	2.164 (1.416-3.308)	<0.0001
T1/T2	150	Reference			
T3/T4	998	4.450 (3.083-6.424)			
Nodal metastasis			<0.0001	0.788 (0.571-1.088)	0.148
N0	377	Reference			
N1-N3	771	2.545 (2.106-3.075)			
Distant metastasis			<0.0001	2.259 (1.785-2.858)	<0.0001
M0	1003	Reference			
M1	145	3.544 (2.906-4.321)			
TNM stage			<0.0001	2.234 (1.596-3.127)	<0.0001
I/II	486	Reference			
III/ IV	662	3.062 (2.570-3.649)			
Lymphovascular invasion			<0.0001	1.393 (1.150-1.688)	0.001
Absent	744	Reference			
Present	404	2.046 (1.749-2.394)			

^*^Cox regression model; ^†^Preoperative serum CEA was measured in 948 patients; ^‡^Preoperative serum CA19-9 was measured in 902 patients; HR indicates hazards ratio; CI indicates confidence interval; CEA indicates carcinoembryonic antigen; CA19-9 indicates carbohydrate antigen 19-9.

## Discussion

The presence of LVI, a common pathological finding for a variety of different cancer types, has been of considerable interest in the last few decades as a potential biomarker. The effectiveness of LVI as a reliable indicator of cancer recurrence and prognosis has been clearly established for both hepatocellular carcinoma and testicular cancer, supporting its incorporation into the UICC/AJCC TNM staging system [19,20]. Previous studies have also shown that the presence of LVI correlated with a poor prognosis. However, due to the lack of large, well-designed and prospective studies, at this time LVI is only recommend to be included in final pathological reports rather than being included in the initial TNM staging system of GC as stated in the NCCN Guidelines for Gastric Cancer of 2013 [21].

In this large-scale retrospective study, through the use of H & E staining, LVI was determined to be present in resected GC specimens at a fairly high frequency. Its presence was also shown to correlate with a higher chance of cancer recurrence and was shown to be an independent predictor of a poor survival rate in post-surgical GC patients.

The presence of LVI was detected in 35.2% of GC patients by H & E staining in this study. Similarly, del Casar et al. had previously reported that 31.9% of GC patients had presented with LVI as detected using H & E staining complemented by immunostaining with CD34 [22]. However, a study by Kim et al. had indicated that LVI was detected in 44.3% of GC patients by immunostaining with D2-40 and CD31 [16]. The differences in the detection rate of LVI could be due to variations in the detection methods. The use of H & E staining, an elastic fiber stain and immunostaining are currently accepted methods in the literature for the detection of LVI. Histological identification of LVI using H & E staining can be subjective, which could lead to the underestimation of the incidence of LVI. However, successful vessel identification using H & E staining has been previously shown to be sufficiently reliable. With quality control measures in place, the prognostic value of LVI as detected by H & E staining was determined for upper urinary tract urothelial carcinoma, breast cancer, colorectal cancer and non-small cell lung cancer [11,23-25]. Additionally, a previous study indicated that both LVI and BVI, as detected by both H & E and IHC staining, significantly correlated with lymph node metastasis [17]. Consistent with previous findings, our data demonstrates that the presence of LVI, as detected by H & E staining, significantly correlates with DFS and DSS in post-surgical GC patients.

Several small-scale studies have previously noted the prognostic value of LVI on DSS and DFS in GC patients. The presence of LVI was shown to be significantly associated with a poorer OS in 77 patients with primary gastric adenocarcinoma [22]. The three-year OS and three-year DFS of 149 GC patients were found to be significantly higher in GC patients without LVI as compared to those with LVI [16]. A retrospective analysis indicated that the OS of the LVI-positive patients, out of 436 stage II GC patients, was shown to be worse than that of the LVI-negative patients [26]. Similarly, we confirmed the negative impact of LVI on DSS and DFS in a large cohort of 1148 patients with gastric adenocarcinoma who underwent gastrectomy. Additionally, a stage-stratified survival analysis determined that the presence of LVI in GC patients correlated with a poorer prognostic outcome. Notably, our study identified LVI as an independent prognostic factor through the use of multivariate analysis. Our findings are in agreement with the results of previously published studies [18,26]. However, it is of note to point out that LVI was not identified as an independent prognostic factor in GC patients in all of the previous studies identified. Kim et al. had reported that the presence of LVI was shown to have a significant impact on patient survival; however, it was not determined to be an independent prognostic factor in GC. A close relationship between the presence of LVI and tumor progression was speculated to be the basis for this negative result [16].

This study, along with previous reports, supports the view that the presence of LVI in GC is a promising indicator of tumor aggressiveness; providing additional information regarding the risk of cancer recurrence and mortality. The addition of LVI assessment to the current UICC/AJCC TNM staging system may lead to a more accurate risk stratification of affected patients and may lead to more appropriate clinical decision-making. Interestingly, randomized controlled trials have recently demonstrated treatment benefits from adjuvant therapy given to GC patients who have undergone surgery [4,27,28]. This supports the idea that GC patients with LVI may be good candidates for further adjuvant therapies that may improve their chances at survival.

The status of nodal metastasis was not evaluated as a statistically significant prognostic factor in multivariate analysis in the present study. However, nodal metastasis was found to be closely correlated with a poor prognosis in our univariate analysis on patient survival. Lymph node status, TNM stage and LVI were included in our multivariate analyses for DSS and DFS. It is known that the status of nodal metastasis is included in TNM staging for GC and there is a strong association between nodal metastasis status and TNM stage. Meanwhile, in agreement with previously published studies, our data indicate that the status of nodal metastasis significantly correlated with the presence of LVI in GC [12,22]. Therefore, the effect of covariate mainly contributes to this negative result.

Consistent with previous studies, the 5-year DSS rate in this study was determined to be approximately 51.0% for all stages, 70.5% for stage I-II and 36.1% for stage III-IV [29,30]. However, the published SEER data indicated that in the United States, the 5-year relative survival for GC was 28.3% for all stages, 64.1% for a localized stage, 28.8% for a regional stage. In fact, it has been suggested that patients with GC have a more favorable prognosis in Asia as compared to those in Europe and the US; a variety of potential reasons have been proposed to explain this. First, the survival advantage of the Asian ethnicity continues to play a role even after being controlled for using other well-known prognostic factors [31,32]. Additionally, the higher surgical quality may contribute to the increased survival rate in Asia; gastrectomy with D2 lymphadenectomy is the standard treatment for GC patients in China. Several clinical trials also have also confirmed the survival benefit for D2 lymph node dissection [33]. Meanwhile, the high incidence of GC in China has subsequently resulted in highly experienced surgeons due to the vast number of times they perform that particular surgery.

Several limitations that could affect the interpretation of our results exist due to the retrospective nature of the study. Potential bias was minimized through the use of strict inclusion and exclusion criteria for patient selection as well as duplicate reviews for each pathologic evaluation carried out according to the commonly used unified international criteria. Further validation of our results will require subsequent large-scale prospective studies.

## Conclusions

Routine H & E staining to determine LVI could be an effective tool in the identification of GC patients that are at an increased risk of tumor recurrence and/or progression. This could also aid in the selection of the appropriate treatment for each patient depending on their status; such as favoring adjuvant therapies in patients with LVI.

## Abbreviations

LVI: Lymphovascular invasion
GC: Gastric cancer
OS: Overall survival
DFS: Disease-free survival
DSS: Disease-specific survival
UICC/AJCC: The International Union Against Cancer/American Joint Committee on Cancer
